# SARS-CoV-2 nsp12 attenuates type I interferon production by inhibiting IRF3 nuclear translocation

**DOI:** 10.1038/s41423-020-00619-y

**Published:** 2021-02-26

**Authors:** Wenjing Wang, Zhuo Zhou, Xia Xiao, Zhongqin Tian, Xiaojing Dong, Conghui Wang, Li Li, Lili Ren, Xiaobo Lei, Zichun Xiang, Jianwei Wang

**Affiliations:** 1 NHC Key Laboratory of System Biology of Pathogens, Institute of Pathogen Biology, Chinese Academy of Medical Sciences & Peking Union Medical College, Beijing, 100730 PR China; 2 Key Laboratory of Respiratory Disease Pathogenomics, Chinese Academy of Medical Sciences and Peking Union Medical College, Beijing, 100730 PR China; 3 Christophe Merieux Laboratory, Institute of Pathogen Biology, Chinese Academy of Medical Sciences & Peking Union Medical College, Beijing, 100730 PR China; 4 Biomedical Pioneering Innovation Center, Beijing Advanced Innovation Center for Genomics, Peking University Genome Editing Research Center, School of Life Sciences, Peking University, Beijing, 100871 China

**Keywords:** SARS-CoV-2, COVID-19, Nsp12, Antiviral immunity, Viral infection, Immune evasion

## Abstract

SARS-CoV-2 is the pathogenic agent of COVID-19, which has evolved into a global pandemic. Compared with some other respiratory RNA viruses, SARS-CoV-2 is a poor inducer of type I interferon (IFN). Here, we report that SARS-CoV-2 nsp12, the viral RNA-dependent RNA polymerase (RdRp), suppresses host antiviral responses. SARS-CoV-2 nsp12 attenuated Sendai virus (SeV)- or poly(I:C)-induced IFN-β promoter activation in a dose-dependent manner. It also inhibited IFN promoter activation triggered by RIG-I, MDA5, MAVS, and IRF3 overexpression. Nsp12 did not impair IRF3 phosphorylation but suppressed the nuclear translocation of IRF3. Mutational analyses suggested that this suppression was not dependent on the polymerase activity of nsp12. Given these findings, our study reveals that SARS-CoV-2 RdRp can antagonize host antiviral innate immunity and thus provides insights into viral pathogenesis.

## Introduction

Coronavirus disease 2019 (COVID-19) has evolved into a global pandemic. According to the WHO Coronavirus Disease dashboard, there were 63,360,234 confirmed cases of COVID-19, including 1,475,825 deaths, as of 2 December 2020 (https://covid19.who.int/). Severe acute respiratory syndrome coronavirus 2 (SARS-CoV-2) has been identified as the pathogenic agent of COVID-19.^1–3^ The International Committee on Taxonomy of Viruses has classified SARS-CoV-2 into the *Coronaviridae* family, the *Orthocoronavirinae* subfamily, the *Betacoronaviruses* genus, and the *Sarbecovirus* subgenus.^4^

SARS-CoV-2 is a positive-stranded RNA virus with a genome of ~29.7 kb.^1^ The replicative cycle of the coronavirus initiates with the translation of ORF1a and ORF1b after entry and genome release^5^. Two large replicase polyproteins (pp1a and pp1ab) are first synthesized. They are then cleaved into 16 nonstructural proteins (nsps), including nsp12, a viral RNA-dependent RNA polymerase (RdRp), by papain-like protease (nsp3) and 3C-like protease (nsp5).^5^ Viral nsps leverage the host cell membrane structure to assemble into replication and transcription complexes that engage in minus-strand RNA synthesis.^5,6^ Subgenomic RNAs are then synthesized, and the structural and accessory proteins are expressed.

Nsp12 consists of 932 amino acids. The structure of nsp12 contains a nidovirus RdRp-associated nucleotidyltransferase (NiRAN) domain and a right-hand RdRp domain.^7^ The NiRAN domain and RdRp domain are connected by an interface domain.^7^ The RdRp domain contains catalytic residues (amino acids 759–761 [SDD]).^7^ Of note, SARS-CoV-2 RdRp has a GDD module (amino acids 823–825), whose residues function as catalytic residues in other viral RdRps, such as HCV NS5b and poliovirus 3D^pol^.^8,9^ In addition to RNA synthesis activity, RdRps of enteroviruses, HCV, tick-borne encephalitis virus, and Langat virus regulate the host innate immune response through inhibition or activation of the expression of IFN-β or other cytokines.^10–13^ In a screen for SARS-CoV-2 genes involved in modulating the host antiviral response,^14^ we identified that nsp12 could inhibit SeV-induced IFN-β promoter activation, but the underlying mechanism is not clear.

The innate immune system is critical for the initial detection and restriction of virus infections. After a cell is infected by a pathogen, pattern recognition receptors, such as RIG-I-like receptors (RLRs) and Toll-like receptors, recognize pathogen-associated molecular patterns (PAMPs) immediately to activate the innate immune system.^15–18^ Retinoic acid-inducible gene I (RIG-I) and melanoma differentiation gene 5 (MDA5) are two cytoplasmic RLRs that recruit the adaptor protein mitochondrial antiviral signaling protein (MAVS) after PAMP recognition.^19–22^ MAVS then initiates signaling pathways involving multiple kinases, which lead to the phosphorylation of interferon regulatory factor 3/7 (IRF3/7).^23^ After phosphorylation and dimerization, IRF3/7 translocates to the nucleus to promote the production of IFN-α/β.^24,25^

Here, we show that SARS-CoV-2 nsp12 attenuates type I IFN responses by inhibiting IRF3 nuclear translocation. To our knowledge, this is the first demonstration that coronavirus RdRp can antagonize type I IFN responses in addition to exhibiting its polymerase and nucleotidyltransferase activities.

## Results

### SARS-CoV-2 nsp12 attenuates type I IFN activation

**Fig. 1:**
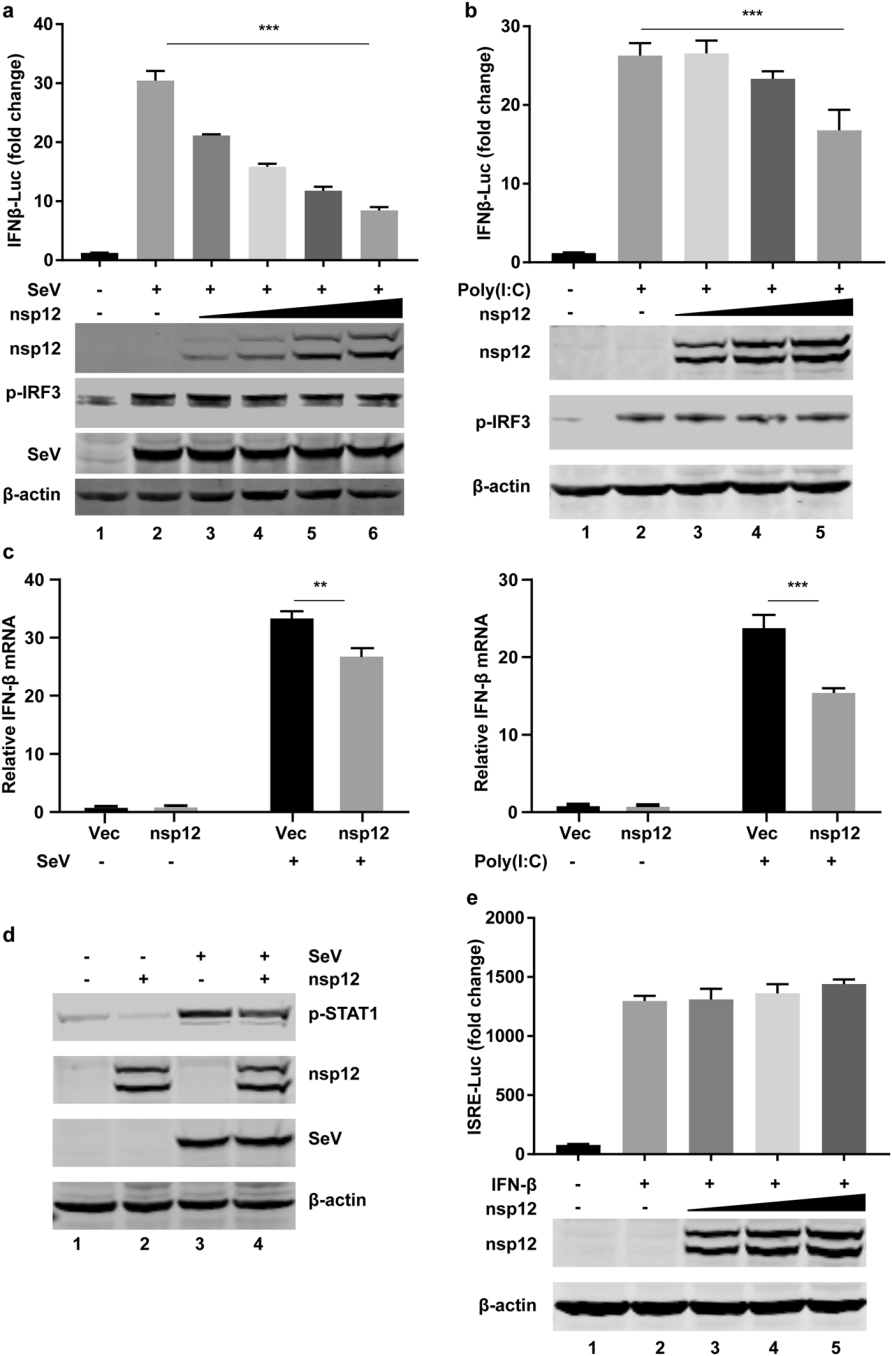
SARS-CoV-2 nsp12 attenuates viral RNA-related type I interferon responses. **a** Effects of nsp12 on SeV-induced IFN-β promoter activation. HEK293T cells were transfected with an IFN-β reporter plasmid along with a control plasmid or with increasing amounts of plasmids expressing nsp12. The cells were infected with SeV for 12 h and assayed for luciferase activity. The indicated protein expression levels were analyzed by western blotting. p-IRF3 was used to assess the stimulation after SeV infection. **b** Effects of nsp12 on high-molecular weight poly(I:C)-induced IFN-β promoter activation. HEK293T cells were transfected with an IFN-β reporter plasmid along with a control plasmid or with increasing amounts of plasmids expressing nsp12. The cells were transfected with high-molecular weight poly(I:C) for 12 h and assayed for luciferase activity. The indicated protein expression levels were analyzed by western blotting. p-IRF3 was used to assess the stimulation of poly I:C treatment. **c** Effect of nsp12 on endogenous *IFN-β* mRNA expression induced by SeV (left) and high-molecular weight poly(I:C) (right). HEK293T cells were transfected with a control plasmid or a plasmid expressing nsp12. After 24 h, cells were infected with SeV for 8 h or transfected with high-molecular weight poly(I:C) for 6 h. Total RNA was extracted, and the expression of *IFN-β* was detected by real-time RT-PCR. **d** Effect of nsp12 on endogenous p-STAT1 induced by SeV. HEK293T cells were transfected with a control plasmid or a plasmid expressing nsp12. After 24 h, the cells were infected with SeV for 8 h. Whole-cell lysates were analyzed by western blotting for p-STAT1, nsp12, and P protein of SeV. **e** Effects of nsp12 on IFN-induced ISRE promoter activation. HEK293T cells were transfected with an ISRE-Luc reporter plasmid along with a control plasmid or with increasing amounts of plasmids expressing nsp12. After 24 h, the cells were treated with IFN (1000 U/ml) for 12 h and assayed for luciferase activity. The indicated protein expression levels were analyzed by western blotting. All experiments were performed at least twice, and one representative is shown. The error bars indicate the SDs of technical triplicates. One-way ANOVA (or the nonparametric equivalent) was used for column analyses (**a**, **b**). The two-tailed unpaired *t-*test was used for two-group comparisons (**c**). **P < 0.01, ***P < 0.001

To examine whether SARS-CoV-2 nsp12 could regulate innate immune responses, we first evaluated the effect of nsp12 on IFN-β promoter activation. 293T cells were transiently transfected with a vector plasmid or plasmids expressing nsp12 along with an IFN-β promoter-driven luciferase reporter plasmid (pIFN-β-Luc) and a control pRL-TK plasmid. After 24 h, the cells were stimulated with SeV or high-molecular weight poly(I:C) (HMW-poly[I:C]), which activates the RIG-I and MDA5 signaling pathways, respectively.^18,24^ The luciferase activity was determined 12 h post stimulation. We found that nsp12 inhibited SeV- or HMW-poly(I:C)-induced IFN-β promoter activation in a dose-dependent manner (Fig. 1a, b). Moreover, this inhibition was observed when the endogenous expression of IFN-β was examined by RT-PCR assays, verifying that nsp12 inhibits the RIG-I and MDA5 signaling pathways (Fig. 1c). Overexpression of nsp12 also inhibited SeV-stimulated STAT1 phosphorylation, an event following IFN-β production (Fig. 1d). We further investigated whether nsp12 affects signaling pathways downstream of IFN-β production. 293T cells were transiently transfected with a vector plasmid or with plasmids expressing nsp12 along with an interferon-stimulated response element (ISRE) reporter plasmid and a control pRL-TK plasmid. After 24 h, the cells were stimulated with IFN-β for 12 h, and the luciferase activity was determined. We found that nsp12 could not inhibit IFN-β-induced ISRE promoter activation (Fig. 1e), suggesting that nsp12 perturbs IFN-β activation rather than downstream signaling.

### SARS-CoV-2 nsp12 inhibits IRF3-triggered IFN-β activation

**Fig. 2:**
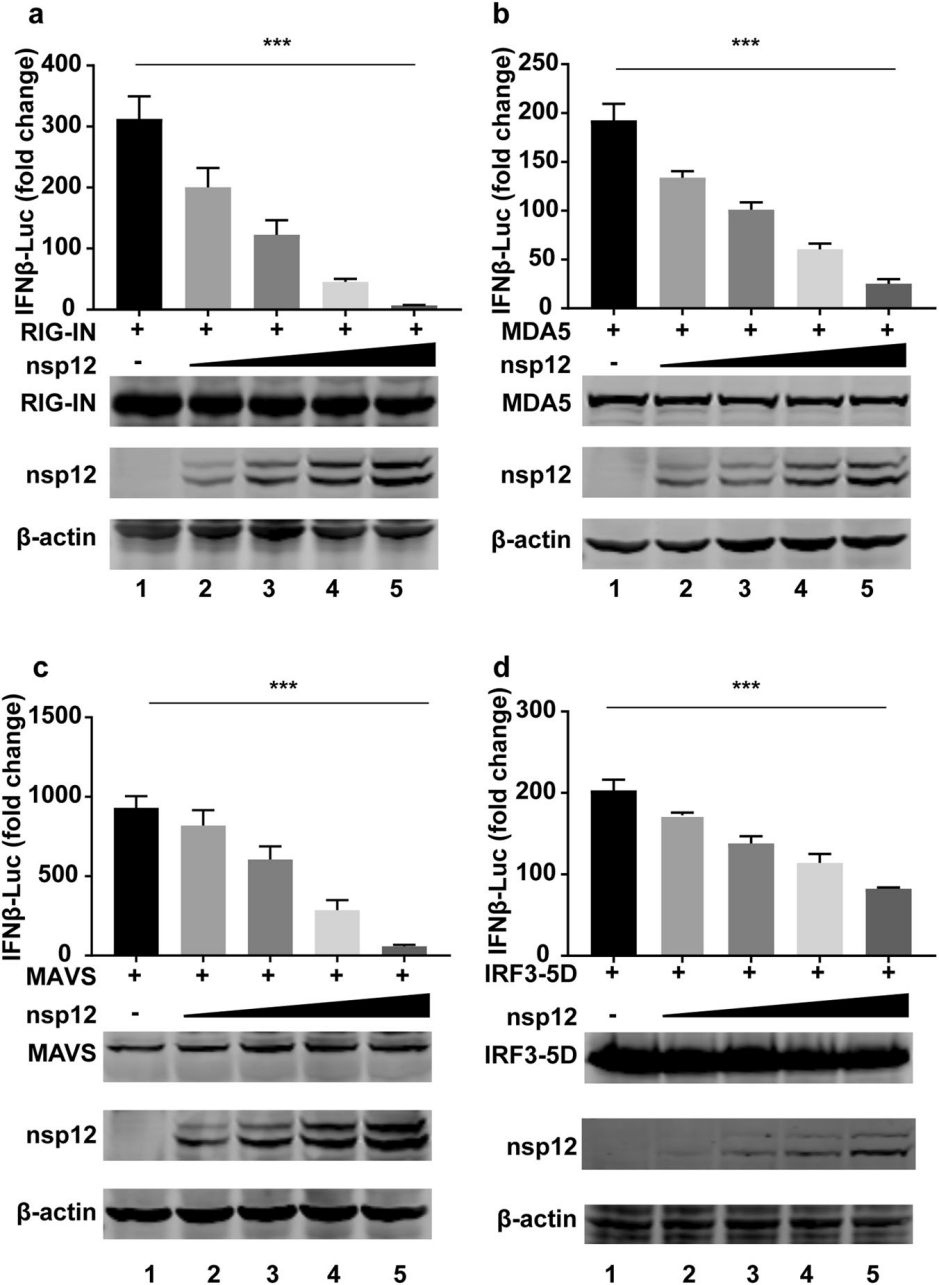
SARS-CoV-2 nsp12 inhibits IRF3 activation. Effects of nsp12 on RIG-IN-, MDA5-, MAVS-, or IRF3-induced IFN-β promoter activation. HEK293T cells were transfected with an IFN-β reporter plasmid along with a control plasmid or with increasing amounts of plasmids expressing nsp12 together with plasmids expressing RIG-IN (**a**), MDA5 (**b**), MAVS (**c**), or IRF3–5D (**d**). At 24 h post transfection, luciferase activity was measured. The expression levels of the indicated proteins were analyzed by western blotting. All experiments were performed at least twice, and one representative is shown. The error bars indicate the SDs of technical triplicates. One-way ANOVA (and the nonparametric equivalent) was used for column analyses, ***P < 0.001

To identify the host targets on which SARS-CoV-2 nsp12 exerts its inhibitory effect, we cotransfected increasing amounts of nsp12 with RIG-IN (an active form of RIG-I), MDA5, MAVS, and IRF3–5D (a constitutively active IRF3 mutant) and determined the activation of the IFN-β promoter. Luciferase reporter assays showed that overexpression of nsp12 inhibited RIG-IN-, MDA5-, MAVS-, and IRF3–5D-triggered IFN-β promoter activation in a dose-dependent manner (Fig. 2a–d). These results demonstrated that nsp12 inhibited IFN-β production at the level of or downstream of IRF3 activation.

### SARS-CoV-2 nsp12 does not inhibit the phosphorylation of IRF3

**Fig. 3:**
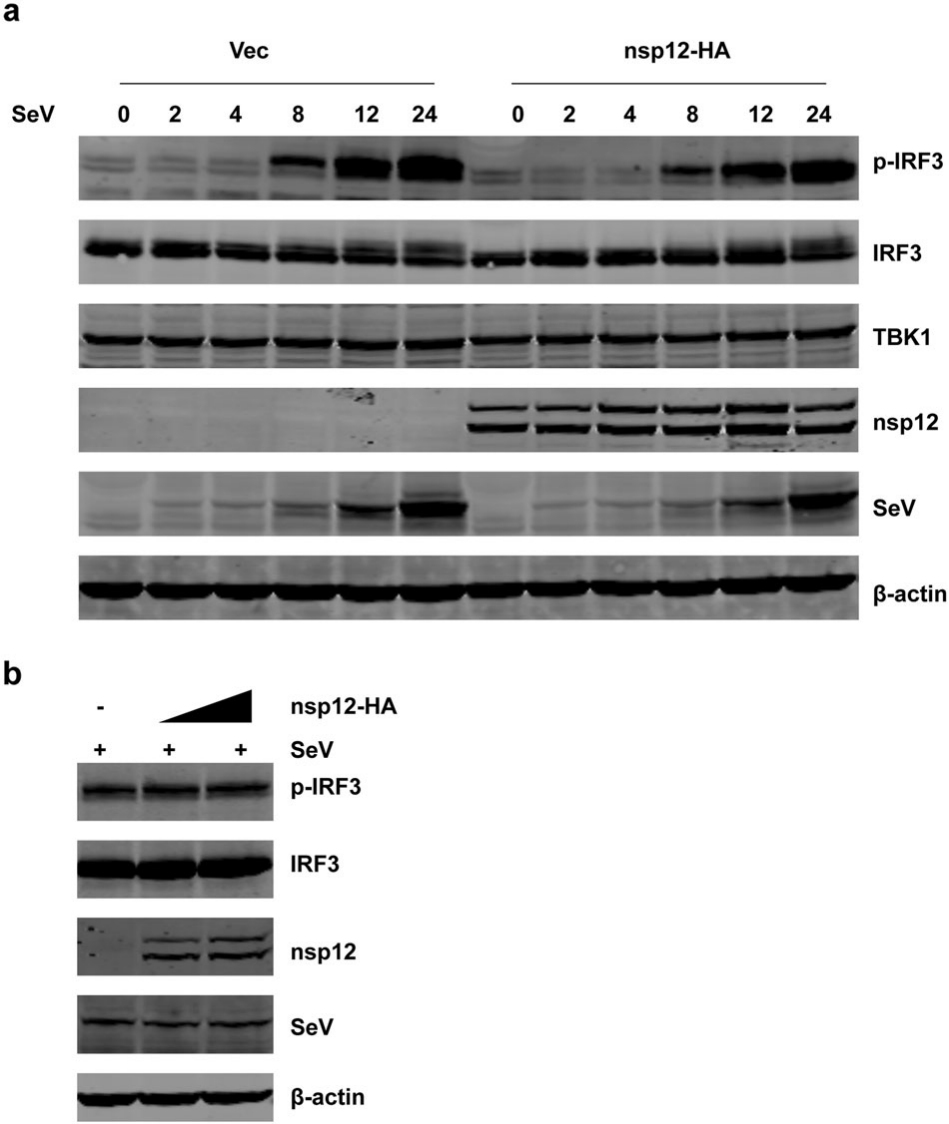
SARS-CoV-2 nsp12 does not inhibit the phosphorylation of IRF3. HEK293T cells were transfected with a control plasmid or nsp12 expression plasmid. After 24 h, the cells were infected with SeV and collected at the indicated times. Whole-cell lysates were analyzed by western blotting for p-IRF3, IRF3, TBK1, nsp12, and P protein of SeV. **b** HEK293T cells were transfected with a control plasmid or increasing amounts of plasmids expressing nsp12. After 24 h, the cells were infected with SeV for 8 h. Whole-cell lysates were analyzed by western blotting for p-IRF3, IRF3, nsp12, and P protein of SeV

IRF3 is activated after phosphorylation by the kinases IKKi and TBK1.^23^ To determine whether SARS-CoV-2 nsp12 antagonizes IRF3 phosphorylation, 293T cells were transiently transfected with a vector plasmid or with plasmids expressing nsp12. After 24 h, the cells were stimulated with SeV for different durations (Fig. 3a). Alternatively, cells were transfected with increasing amounts of plasmids expressing nsp12 for 24 h and were then infected with SeV for 8 h (Fig. 3b). Sev induced IRF3 phosphorylation at comparable levels in the presence or absence of nsp12 (Fig. 3a, b) in various settings, suggesting that SARS-CoV-2 nsp12 does not affect virus-triggered IRF3 phosphorylation.

### SARS-CoV-2 nsp12 suppresses the nuclear translocation of IRF3

**Fig. 4:**
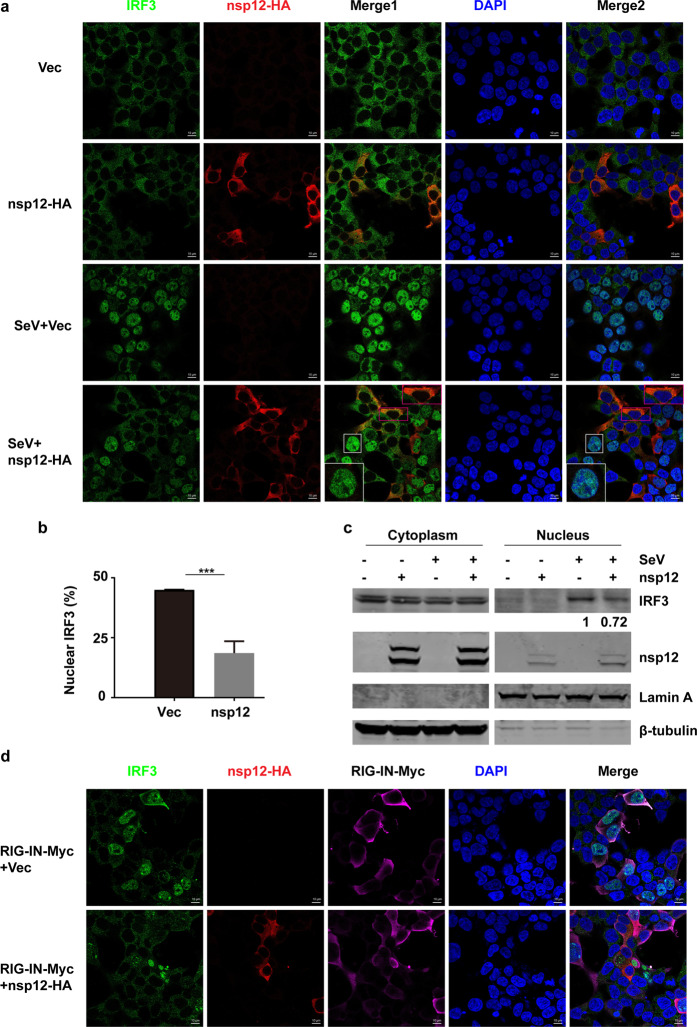
SARS-CoV-2 nsp12 suppresses the nuclear translocation of IRF3. **a** Effect of SARS-CoV-2 nsp12 on SeV-induced nuclear translocation of IRF3. HEK293T cells were transfected with a control plasmid or an nsp12 expression plasmid. At 24 h post transfection, the cells were infected with SeV for 4 h and then immunostained with the indicated antibodies. Merge 1 and Merge 2 indicate the merged red and green channels and the merged red, green, and blue channels, respectively. Scale bar, 10 μM. The enlarged image is a magnified view of the corresponding box. **b** Quantitation of the nuclear translocation of IRF3. The percentage of nuclear IRF3-positive transfected cells was determined in three independent experiments. The two-tailed unpaired *t-*test was used for two-group comparisons, ***P < 0.001. **c** Immunoblot analysis of cytoplasmic and nuclear IRF3 after SeV infection. HEK293T cells were transfected with a control plasmid or an nsp12 expression plasmid. After 24 h, the cells were infected with SeV for 4 h and fractionated into cytoplasmic and nuclear fractions. The fractions were analyzed by western blotting for IRF3 and nsp12 detection. β-Tubulin and Lamin A were used as a cytoplasmic and a nuclear marker, respectively. The numbers under the lanes indicate the IRF3 band intensity, which was normalized to that of Lamin A. **d** Effect of SARS-CoV-2 nsp12 on RIG-IN-induced nuclear translocation of IRF3. HEK293T cells were transfected with RIG-IN or RIG-IN and nsp12 expression plasmids. At 24 h post transfection, the cells were immunostained with the indicated antibodies

The translocation of phosphorylated IRF3 into the nucleus is critical for IFN-β transcription. Since nsp12 does not affect IRF3 phosphorylation, we then examined whether nsp12 affects IRF3 nuclear translocation in 293T cells. Immunofluorescence analyses showed that in uninfected cells, IRF3 was distributed in the cytoplasm with or without overexpression of nsp12 (Fig. 4a, row 1 and 2). In cells transfected with control vectors, IRF3 was efficiently translocated to the nucleus upon SeV infection (Fig. 4a, row 3). However, SeV-induced IRF3 nuclear translocation was severely impaired in cells expressing nsp12 (Fig. 4a (row 4), b). Similar results were observed in HeLa–ACE2 cells that are permissive to SARS-CoV-2 infection (Supplementary Fig. 1). Nuclear fractionation assays further confirmed this observation, showing that nsp12 overexpression substantially suppressed SeV-triggered nuclear accumulation of IRF3 (Fig. 4c). These data support that SARS-CoV-2 nsp12 can inhibit IRF3 nuclear translocation in response to virus infection. Moreover, this nsp12-mediated inhibition was recapitulated when IRF3 nuclear translocation was stimulated by RIG-IN overexpression (Fig. 4d).

We then investigated whether nsp12 exerts its inhibitory effect by physically interacting with IRF3. Coimmunoprecipitation assays showed that nsp12 did not associate with IRF3 in the presence or absence of SeV infection (Supplementary Fig. 2a). Serving as the positive control, TBK1 was efficiently coimmunoprecipitated with IRF3 (Supplementary Fig. 2b).

### Nsp12 inhibits IFN-β production via a mechanism independent of its enzymatic activity

**Fig. 5:**
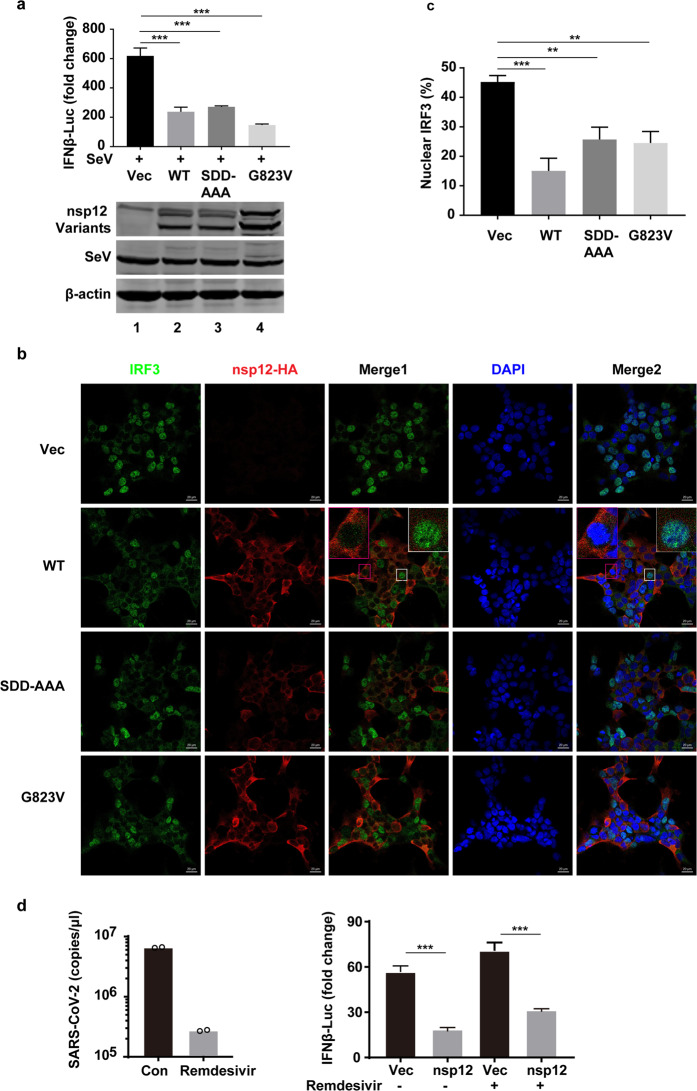
The polymerase activity of SARS-CoV-2 nsp12 is not required for suppression of type I IFN activation. **a** Effects of SARS-CoV-2 nsp12 and its mutants on SeV-induced IFN-β promoter activation. HEK293T cells were transfected with an IFN-β reporter plasmid along with a control plasmid or plasmids expressing wild-type SARS-CoV-2 nsp12 or the indicated SARS-CoV-2 nsp12 variants. The cells were infected with SeV for 12 h and assayed for luciferase activity. The expression levels of the indicated proteins were analyzed by western blotting. **b** Effect of SARS-CoV-2 nsp12 and its mutants on SeV-induced nuclear translocation of IRF3. HEK293T cells were transfected with a control plasmid or plasmids expressing wild-type SARS-CoV-2 nsp12 or the indicated SARS-CoV-2 nsp12 variants. At 24 h post transfection, the cells were infected with SeV for 4 h and then immunostained with the indicated antibodies. Merge 1 and Merge 2 indicate the merged red and green channels and the merged red, green, and blue channels, respectively. Scale bar, 20 μM. The enlarged image is a magnified view of the corresponding box. **c** Quantitation of the nuclear translocation of IRF3. **d** Vero cells were infected with SARS-CoV-2 at an MOI of 0.1 with or without 10 μM remdesivir for 24 h. The viral copies in the supernatants were measured using real-time RT-PCR (left panel). HEK293T cells were transfected with an IFN-β reporter plasmid along with a control plasmid or nsp12 expression plasmid and a plasmid expressing IRF3–5D. Remdesivir (10 μM) was added to the culture medium at 5 h post transfection. At 24 h post transfection, luciferase activity was measured (right panel). All experiments were performed at least twice, and one representative is shown. The error bars indicate the SDs of technical triplicates. The two-tailed unpaired *t-*test was used for two-group comparisons compared to the vector group, **P < 0.01, ***P < 0.001

As nsp12 is responsible for catalyzing the synthesis of viral RNA, we explored whether its enzymatic activity is involved in IFN regulation. First, we investigated whether the RdRp activity of SARS-CoV-2 nsp12 is required for inhibiting the nuclear translocation of IRF3 upon SeV infection. Two nsp12 variants harboring mutations in the putative RdRp catalytic residues found in coronavirus and other RNA viruses, including SDD-AAA (in which amino acids 759–761 are substituted with alanine residues) and G823V (in which amino acid 823 is substituted with valine), were generated. The effects of wild-type nsp12 and nsp12 mutants on IFN promoter activation and the nuclear translocation of IRF3 were then assayed. We found that the SDD-AAA and G823V mutants of nsp12 suppressed SeV-induced IFN-β promoter activation to a level comparable to that of wild-type nsp12 (Fig. 5a). These two mutants also suppressed the nuclear translocation of IRF3 upon SeV infection (Fig. 5b, c).

To further examine whether polymerase activity is associated with nsp12’s antagonistic function, we tested the effect of remdesivir, a nucleoside analog antiviral drug that inhibits the polymerase activity of nsp12.^7^ Remdesivir efficiently inhibited SARS-CoV-2 replication in cell culture (Fig. 5d left panel); however, it failed to block nsp12’s antagonistic activity on IFN-β activation triggered by the overexpression of IRF3–5D (Fig. 5d right panel). On the basis of the collective data, we conclude that SARS-CoV-2 nsp12 can suppress IFN-β activation in a polymerase activity-independent manner.

**Fig. 6:**
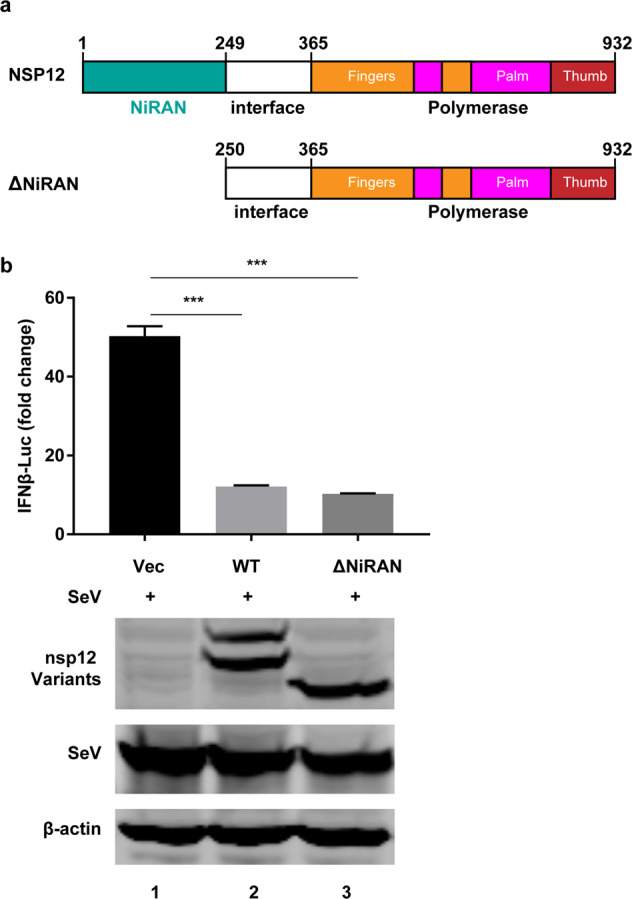
IFN regulation by SARS-CoV-2 nsp12 is not related to the NiRAN domain. **a** Schematic diagram of SARS-CoV-2 nsp12. **b** Effects of SARS-CoV-2 nsp12 and the ΔNiRAN mutant on SeV-induced IFN-β promoter activation. HEK293T cells were transfected with an IFN-β reporter plasmid along with a control plasmid or plasmids expressing wild-type nsp12 or the ΔNiRAN mutant. The cells were infected with SeV for 12 h and assayed for luciferase activity. The expression levels of the indicated protein were analyzed by western blotting. The two-tailed unpaired *t-*test was used for two-group comparisons compared to the vector group, ***P < 0.001

We then examined the effect of the nsp12 NiRAN domain, which is proposed to have nucleotidyltransferase activity. To do this, we constructed a nsp12 mutant devoid of the NiRAN domain (ΔNiRAN). 293T cells were transiently transfected with a vector plasmid or plasmids expressing nsp12 or ΔNiRAN along with pIFN-β-Luc and pRL-TK. After 24 h, the cells were stimulated with SeV for 12 h. We found that the ΔNiRAN mutant exerted an inhibitory effect on IFN comparable to that of wild-type nsp12 (Fig. 6), suggesting that the NiRAN domain is not involved in IFN suppression.

The coronavirus polymerase complex is composed of nsp12 and two cofactors, nsp7 and nsp8.^26^ We next texted whether these two cofactors play roles in nsp12-mediated IFN suppression. We found that although nsp7 and nsp8 are reported to promote the polymerase activity of nsp12,^27^ they failed to potentiate nsp12’s inhibitory activity on IFN (Supplementary Fig. 3), further suggesting that nsp12 functions as an IFN antagonist independently.

## Discussion

SARS-CoV-2 infection induces the production of aberrant type I IFN in cultured cells and COVID-19 patients.^28^ This phenomenon is at least partly due to the various viral mechanisms for evasion and suppression of IFN activation.^29,30^ Several SARS-CoV-2 proteins, including nsp1, nsp3, nsp6, nsp8, nsp12, nsp13, nsp14, nsp15, ORF3, ORF6, ORF8, ORF9b, M, and N, have been reported to inhibit type I IFN activation and the corresponding response.^14,31–39^ Here, we report that nsp12 of SARS-CoV-2 attenuates type I IFN induction by inhibiting IRF3 nuclear translocation.

In our and other groups’ efforts to screen viral proteins that potentially regulate type I IFN,^14,38^ multiple nsps, including nsp12, nsp13, nsp14, and nsp15, have been found to exert antagonistic activities. These nsps are immediately translated from incoming viral genomic RNAs. We postulate that these “early” viral proteins, including nsp12, may help to antagonize the host innate immune response triggered by incoming viral RNAs or other signals at the beginning of infection. These proteins are then recruited to double-membrane vesicles, where they form a replication/transcription complex to catalyze viral genomic replication and transcription. Then, “late” viral proteins are translated from subgenomic RNAs, including ORF3, ORF6, and M, which exert potent inhibitory effects on host IFN activation and signaling.

The polymerases of other RNA viruses have been reported to regulate type I IFN activation. 3D^pol^ of enterovirus 71 and coxsackievirus B3 interact with the caspase activation and recruitment domains of MDA5 to inhibit IFN-β promoter activation and mRNA expression.^10^ Similar to the case for SARS-CoV-2 nsp12, the polymerase activity of EV71 3D^pol^ is not required to suppress type I IFN activation.^10^ Polymerases of flaviviruses such as hepatitis C virus, tick-borne encephalitis virus, and Langat virus regulate the type I IFN response via RdRp activity.^11–13^ These findings indicate that viral polymerase could play pleiotropic roles in addition to exerting RdRp activity. It is unclear how nsp12 inhibits IRF3 nuclear translocation, and the underlying mechanism awaits further investigation.

In summary, we demonstrate that SARS-CoV-2 nsp12 attenuates type I IFN activation by inhibiting IRF3 nuclear translocation. This inhibitory effect is independent of nsp12 polymerase activity. Further understanding of the role and mechanism of action of nsp12 may direct us to novel therapeutic targets.

## Methods

### Cell lines and viruses

Human 293T (ATCC, #CCL-11268), HeLa (ATCC, #CCL-2), and Vero (ATCC, #CCL-81) cells were cultured in Dulbecco’s modified Eagle’s medium (Invitrogen, Carlsbad, CA) supplemented with 10% heat-inactivated fetal bovine serum (FBS) (HyClone, Logan, UT), 100 U/ml penicillin, and 100 U/ml streptomycin at 37 °C in a 5% CO_2_ humidified atmosphere. Low-passage cells were used after direct purchase from ATCC, and all cells were mycoplasma-free. SARS-CoV-2 was isolated from respiratory samples of confirmed COVID-19 patients by inoculation onto Vero cells^1^ and was propagated in Vero cells for use in this study. Cells were infected with SARS-CoV-2 at a multiplicity of infection (MOI) of 0.1 or 0.5. Unbound virus was washed away after 1 h, and cells were then cultured with fresh medium supplemented with 2% FBS. All experiments with SARS-CoV-2 were conducted in the BSL-3 laboratory.

### Plasmids and antibodies

The SARS-CoV-2 nsp12 gene (IPBCAMS-WH-01/2019 strain, no. EPI_ISL_402123) was optimized by Gene Designer 1.0 and cloned into the pCMV6-Entry expression vector with an HA-tag at the C terminus. The plasmids Flag-RIG-IN, Flag-MDA5, HA-MAVS, pGL3-IFN-β–Luc, IRF3–5D-Flag, and pRL-TK have been described elsewhere.^40^ Mutated variants of SARS-CoV-2 nsp12-tagged HA were constructed by using a site-directed mutagenesis kit (Stratagene, La Jolla, CA). All variants were confirmed by subsequent sequencing.

The antibodies used in this research included the following: a SARS-CoV-2 (COVID-19) RdRp (nsp12) antibody from GeneTex (1:1000, Cat#GTX135467), a Flag antibody from Sigma-Aldrich (1:4000, Cat# F3165), a β-actin antibody from Sigma-Aldrich (1:4000, Cat# A5441), an HA antibody from Sigma-Aldrich (1:10000, Cat# H9658); a p-STAT1 antibody from Thermo Fisher (1:1000, Cat# 700349), a TBK1 antibody from Cell Signaling Technology (1:1000, Cat# 3504), an IRF3 antibody from Abcam (1:1000, Cat# 76409) a p-IRF3 antibody (S386) from Abcam (1:1000, Cat# 76493), a Lamin A antibody from Sigma-Aldrich (1:2000, Cat# L1293), a β-tubulin antibody from Zsbio (1:1000, Cat# TA-10), and a SeV antibody from MBL (1:2000, PD029C1). A Dual-Luciferase^®^ Reporter Assay System was purchased from Promega (Madison, WI). IRDye 800-labeled IgG and IRDye 680-labeled IgG secondary antibodies were purchased from LI-COR Biosciences (Lincoln, NE).

### Reporter assays

293T cells cultured in 24-well plates were transfected with luciferase reporter plasmids or plasmids expressing indicated proteins. Cells were harvested, and the cell lysates were used to determine luciferase activity using a Dual Luciferase Reporter Assay System (Promega). The firefly luciferase activity levels were normalized to the Renilla luciferase activity levels.^40^

### Western blot analysis

The samples were separated on denaturing SDS–PAGE gels and transferred electrophoretically onto nitrocellulose membranes. Proteins of interest were immunoblotted with the indicated primary antibodies and IRDye secondary antibodies (LI-COR Biosciences, Lincoln, NE). The protein expression levels were detected by using the Odyssey Infrared Imaging System (LI-COR Biosciences) and analyzed by using the integrated software of the Odyssey system (Image Studio Ver 5.2).

### Immunofluorescence

Cells were washed with PBS and fixed with 4% paraformaldehyde. Then, the cells were permeabilized with 0.5% Triton X-100. After the cells were washed with PBS, they were blocked and stained with primary antibodies before being stained with Alexa Fluor 488-, 594- and 647-conjugated secondary antibodies.^41^ Nuclei were stained with DAPI (Sigma). The antibodies used in this research were an IRF3 antibody from Cell Signaling Technology (1:200, Cat# 11904), an HA antibody from Sigma-Aldrich (1:100, Cat# H9658), and a Myc antibody from Sigma-Aldrich (1:200, Cat# M4439). Fluorescence images were obtained and analyzed using a laser scanning confocal microscope (Zeiss LSM 800).

### Quantitative real-time PCR analysis

Total RNA was extracted by using TRIzol reagent (Invitrogen, Carlsbad, CA) and reverse transcribed into cDNA by M-MLV Reverse Transcriptase (Promega, Madison, WI). The cDNA was prepared for real-time PCR by using TB Green Premix Ex (Takara, Kusatsu, Shiga). The primer sequences used for *IFNβ* are 5′ TAGCACTGGCTGGAATGAG3′ (forward) and 5′GTTTCGGAGGTAACCTGTAAG 3′ (reverse).^41^

### Statistics

One-way ANOVA or the nonparametric equivalent was used for column analyses. The two-tailed unpaired *t-*test was used for two-group comparisons. **P < 0.01, ***P < 0.001.

## Supplementary information

SUPPLEMENTAL INFORMATION

Supplementary Fig. 1

Supplementary Fig. 2

Supplementary Fig. 3

## Data Availability

All data related to this paper may be requested from the authors.
