# Supplementary material for: SARS-CoV-2 nsp12 attenuates type I interferon production by inhibiting IRF3 nuclear translocation
Source: Cell Mol Immunol. 2021 Feb 26;18(4):945–53. doi: 10.1038/s41423-020-00619-y (PMC7907794; doi:10.1038/s41423-020-00619-y)
Supplement: Supplementary file 1 — SUPPLEMENTAL INFORMATION [file 41423_2020_619_MOESM1_ESM.doc]

**SUPPLEMENTAL INFORMATION**

**Supplementary Figure. SARS-CoV-2 nsp12 suppresses the nuclear translocation of IRF3 in HeLa-ACE2 cells.** **a** HeLa-ACE2 cells were infected with SARS-CoV-2 at 0.5 MOI for indicated times. Whole cell lysates were analyzed by Western-blot for N protein of SARS-CoV-2 and ACE2. **b** Effect of SARS-CoV-2 nsp12 on SeV-induced nuclear translocation of IRF3. HeLa**-**ACE2 cells were transfected with control plasmid or nsp12 expression plasmid. At 24h post transfection, cells were infected with SeV for 4h, followed by immunostaining with indicated antibodies. Scale bar, 5 μM.

**Supplementary Figure 2. SARS-CoV-2 nsp12 did not associated with endogenous IRF3. a** HEK293T cells were transfected with control plasmid or plasmid expressing nsp12. After 24h, cells were infected with or without SeV for 4h. Cell lysates were immunoprecipitated (IP) with anti-HA antibody. Immunoprecipitates and aliquots of cell lysates were subjected to Western blot analysis with antibodies against IRF3, HA, P protein of SeV and β-actin. **b** HEK293T cells were transfected with control plasmid or plasmids expressing TBK1-Flag. After 24h, cell lysates were immunoprecipitated (IP) with anti-Flag antibody. Immunoprecipitates and aliquots of cell lysates were subjected to Western blot analysis with antibodies against IRF3, Flag and β-actin.

**Supplementary Figure 3. IFN regulation ability of SARS-CoV-2 nsp12 is independent on its co-factors nsp7 and nsp8.** HEK293T cells were transfected with an IFN-β reported plasmid, along with a control plasmid or with plasmids expressing nsp12, and/or nsp7, nsp8. Cells were infected with SeV for 12 h and assayed for luciferase activity. Indicated protein expressions were analyzed by Western Blot. The two-tailed unpaired *t-*test was used for two-group comparisons, ***P* < 0.01.
