# Supplementary figures and images for: SARS-CoV-2 nsp12 attenuates type I interferon production by inhibiting IRF3 nuclear translocation
Source: Cell Mol Immunol. 2021 Feb 26;18(4):945–53. doi: 10.1038/s41423-020-00619-y (PMC7907794; doi:10.1038/s41423-020-00619-y)

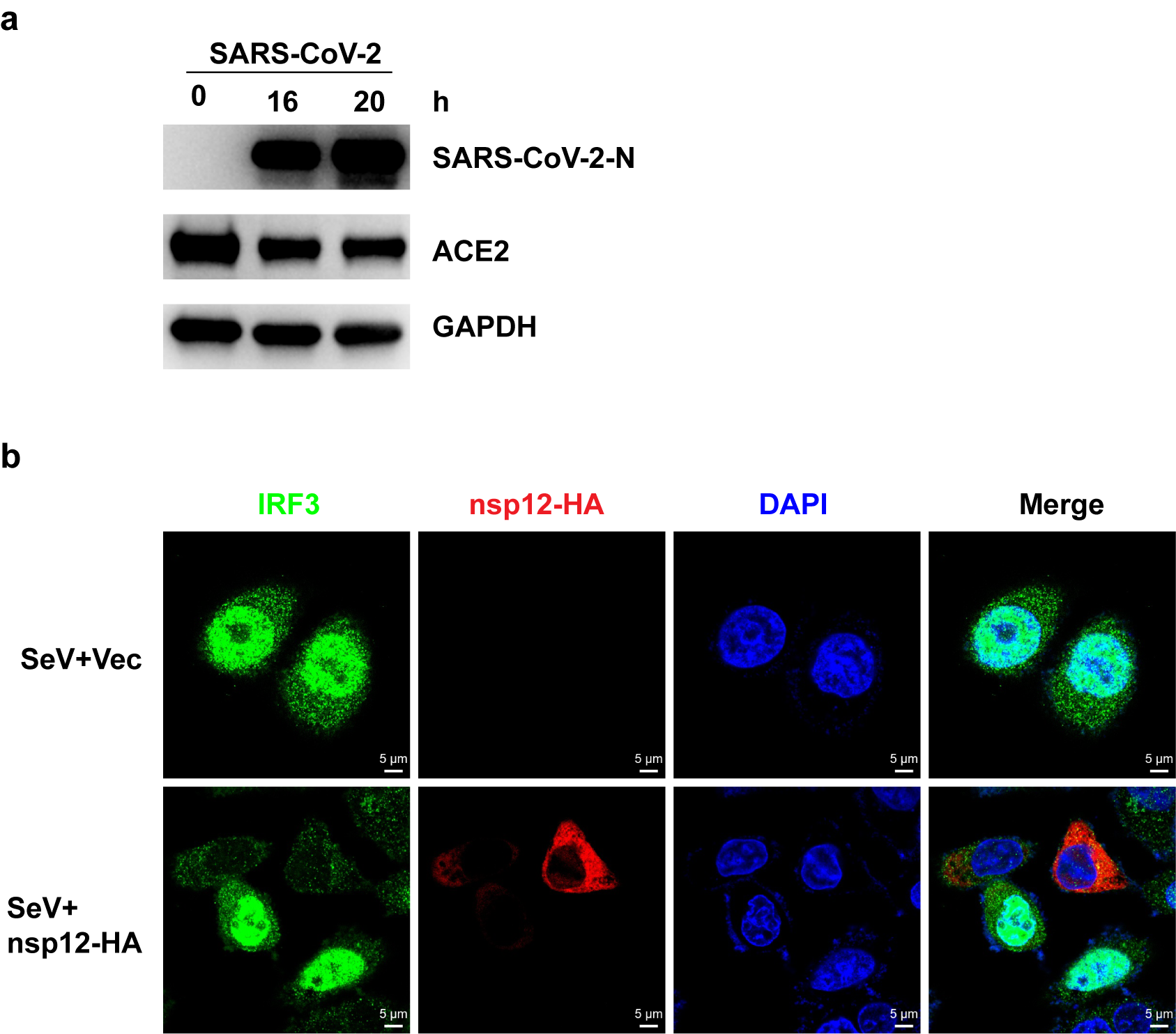

Supplement: Supplementary file 2 — Supplementary Fig. 1 [file 41423_2020_619_MOESM2_ESM.tif]

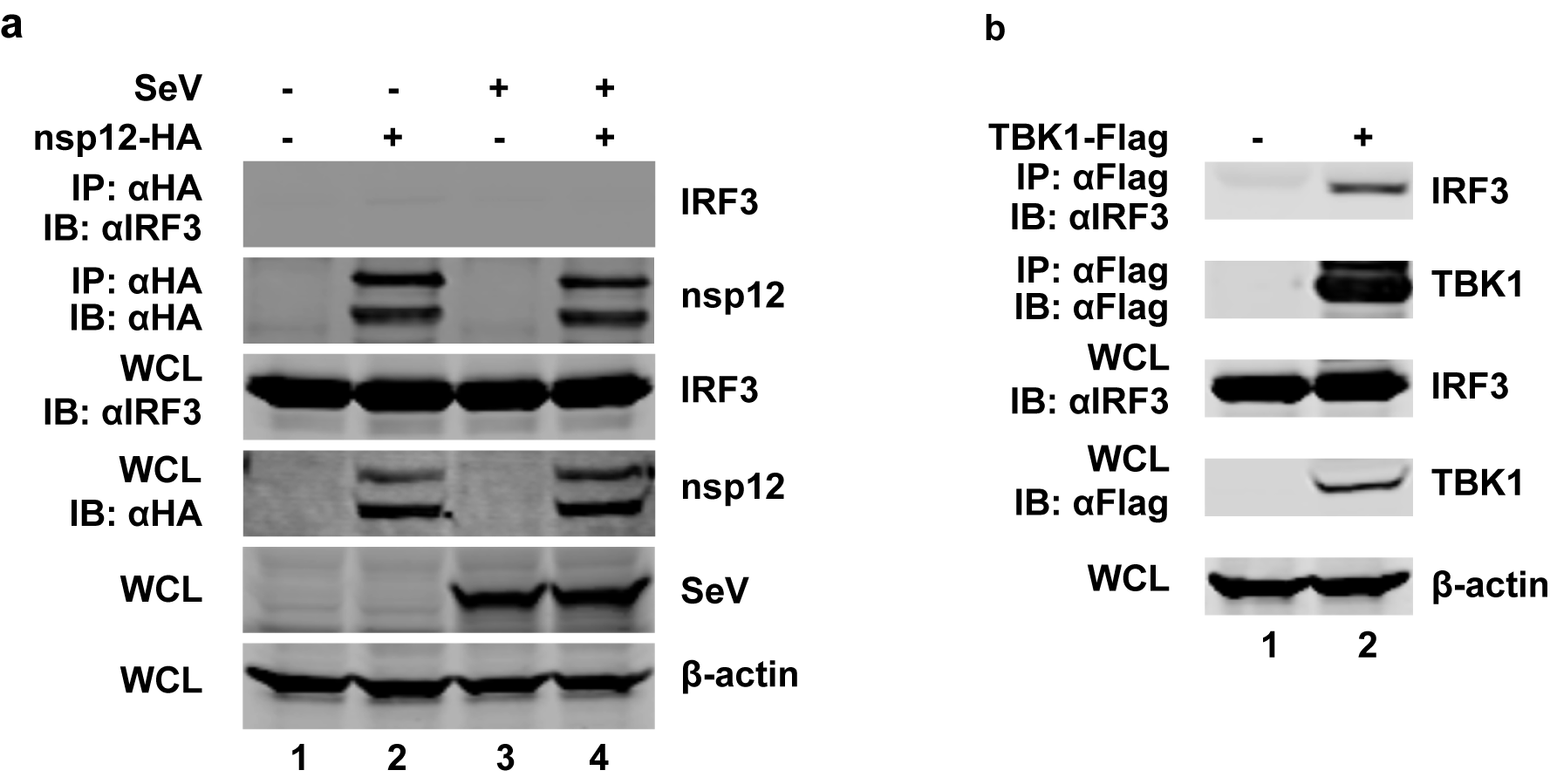

Supplement: Supplementary file 3 — Supplementary Fig. 2 [file 41423_2020_619_MOESM3_ESM.tif]

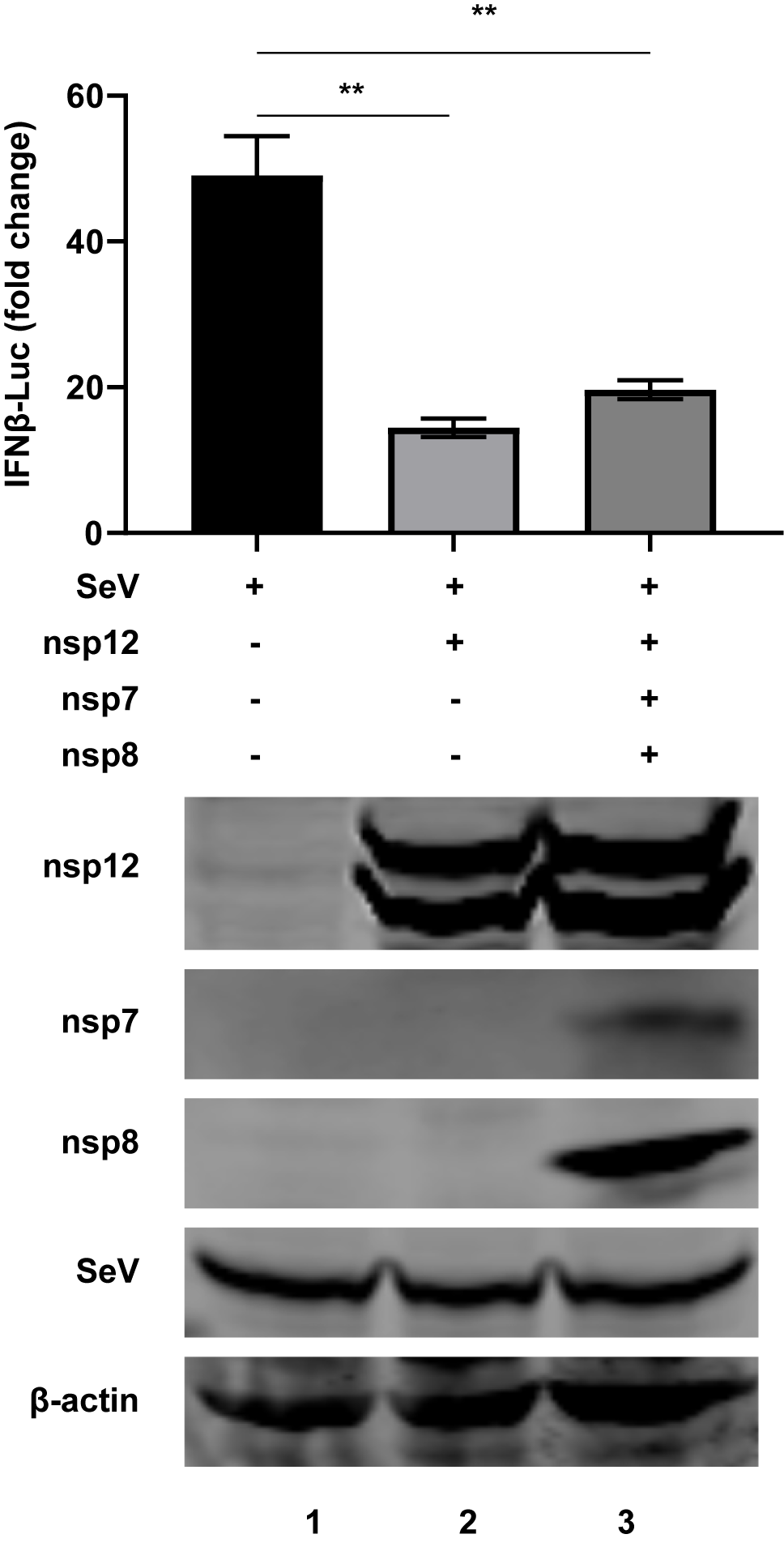

Supplement: Supplementary file 4 — Supplementary Fig. 3 [file 41423_2020_619_MOESM4_ESM.tif]
